# Postoperative hepatitis B virus reactivation and its impact on survival in HBV-related hepatocellular carcinoma patients undergoing conversion therapy with interventional therapy combined with tyrosine kinase inhibitors and immune checkpoint inhibitors

**DOI:** 10.3389/fcimb.2025.1598193

**Published:** 2025-07-17

**Authors:** Shaowei Xu, Qingqing Pang, Meng Wei, Danxi Liu, Du Yuan, Tao Bai, Xiaobo Wang, Zhihong Tang, Feixiang Wu

**Affiliations:** ^1^ Department of Hepatobiliary Surgery, Guangxi Medical University Cancer Hospital, Nanning, Guangxi, China; ^2^ Department of Oncology, Liuzhou Workers’ Hospital, Liuzhou, Guangxi, China

**Keywords:** hepatocellular carcinoma, conversion therapy, surgery, HBV reactivation, survival

## Abstract

**Objective:**

This study aimed to investigate hepatitis B virus (HBV) reactivation and its impact on postoperative survival in patients with HBV-related hepatocellular carcinoma (HCC) who underwent conversion therapy. The therapeutic regimen consisted of interventional procedures (hepatic artery infusion chemotherapy [HAIC] and/or transarterial chemoembolization [TACE]) combined with tyrosine kinase inhibitors (TKIs) and immune checkpoint inhibitors (ICIs).

**Methods:**

A retrospective analysis was performed at a single institution involving 91 patients *who had* initially unresectable HCC linked to the hepatitis B virus. These patients achieved resectability following conversion therapy and subsequently underwent surgical tumor removal. Logistic regression identified risk factors for HBV reactivation (HBVr). Kaplan-Meier survival analysis and log-rank tests assessed survival differences. Cox proportional hazards regression was used to identify independent predictors of progression-free survival (PFS) and overall survival (OS).

**Results:**

In our cohort, HBVr occurred in 17 patients (18.7%), all of whom received antiviral therapy. The incidence of HBVr was 16.7% (14/84) in patients with detectable baseline HBV DNA and 42.9% (3/7) in those with undetectable levels. Baseline HBV DNA ≥2000 IU/ml was identified as an independent protective factor against HBVr (OR 0.090, 95% CI 0.015–0.532; P = 0.008). The median PFS was significantly shorter in the reactivation group than in the non-reactivation group (12.1 months [95% CI 5.5–18.7] *vs*. 29.2 months [95% CI 23.6–34.7]; P < 0.001). However, no significant difference was observed in median OS between the two groups (not reached *vs*. 45.6 months [95% CI 41.7–49.5]; P = 0.117).

**Conclusion:**

HBVr represents a potential complication in subjects receiving hepatectomy for hepatitis B virus associated HCC following conversion therapy involving interventional therapies combined with TKIs and ICIs. Patients experiencing HBVr exhibited significantly shorter progression-free survival compared to those without reactivation. Therefore, prophylactic antiviral therapy and meticulous HBV DNA monitoring are warranted during both conversion therapy and the perioperative period.

## 1 Introduction

Hepatocellular carcinoma (HCC) is a prevalent malignancy worldwide, with approximately 70% of new cases occurring in Asia (Sung et al., 2021). Projections estimate that there will be over one million new HCC cases and related deaths annually by 2040 (Rumgay et al., 2022). In regions with high HCC incidence, hepatitis B virus (HBV) infection is the primary etiological factor (Mysore and Leung, 2018). In recent years, systemic therapy has emerged as the mainstream treatment for advanced HCC. Specifically, the combination of tyrosine kinase inhibitors (TKIs) and immune checkpoint inhibitors (ICIs) has achieved objective response rates (ORRs) of 20–30% in advanced or unresectable HCC, as demonstrated in landmark trials such as IMbrave150, ORIENT-32, HIMALAYA, and CARES-310. Furthermore, combining these systemic agents with locoregional therapies, such as transarterial chemoembolization (TACE) or hepatic arterial infusion chemotherapy (HAIC), has yielded even better outcomes (Ju et al., 2021; Cai et al., 2022; Fu et al., 2023; Zhu et al., 2023). Multiple studies have shown that surgical resection following successful conversion therapy offers superior long-term survival benefits compared to palliative treatments alone (Kulik et al., 2006; Lewandowski et al., 2009; Shindoh et al., 2021). Therefore, for patients with initially unresectable HCC, the selection of optimal treatment strategies and timing, alongside the effective management of complications, is of paramount importance for improving prognosis.

Among these complications, hepatitis B virus reactivation (HBVr) is a well-recognized challenge during HCC treatment (Voican et al., 2016). While HBVr is more frequent in patients positive for hepatitis B surface antigen (HBsAg) and antibody to hepatitis B core antigen (anti-HBc), it can also manifest in individuals with resolved HBV infection (Hoofnagle, 2009). Existing antiviral agents are unable to completely eradicate covalently closed circular DNA (cccDNA), the viral reservoir in patients with chronic hepatitis B. Consequently, when cccDNA persists in the context of immunosuppression, control over HBV replication is compromised, leading to reactivation (Shi and Zheng, 2020). HBVr can trigger a spectrum of clinical events, ranging from mild hepatitis to fulminant liver failure and even death (Papatheodoridis et al., 2022). Moreover, HBVr can necessitate the interruption of anti-tumor therapy and adversely affect overall survival (Yang et al., 2024).

Previous research has reported an elevated risk of HBVr following surgical resection for HCC, which detrimentally affects patient prognosis (Huang et al., 2012; Dan et al., 2013; Xie et al., 2015). HBVr has also been observed during and after various anti-tumor regimens for intermediate-to-advanced HCC, including interventional therapies, TKIs, and ICIs, often leading to severe complications and negatively impacting long-term survival (Shen et al., 2023; Yang et al., 2024). Conversion therapy, the process of transforming an initially unresectable HCC into a resectable state, aims to enhance surgical eligibility and prognosis. Presently, a growing number of patients with unresectable HCC are undergoing triple therapy (interventional therapy plus TKIs and ICIs), which subsequently allows them to receive surgical treatment. However, for this specific population, the incidence of HBVr and its impact on prognosis remain unclear. This retrospective study, therefore, aims to investigate the occurrence of HBVr in HBV-related HCC patients who underwent surgical resection after conversion therapy with interventional treatment plus TKIs and ICIs, and to evaluate its influence on their prognosis.

## 2 Materials and methods

### 2.1 Patient recruitment and study design

This retrospective study enrolled patients with HCC who underwent tumor resection following conversion therapy with HAIC or TACE combined with TKIs and ICIs at the Guangxi Medical University Cancer Hospital from January 2021 to April 2024.The inclusion criteria were as follows: (1) age between 18 and 85 years; (2) histologically confirmed HCC; (3) Barcelona Clinic Liver Cancer (BCLC) stage A–C; (4) chronic or resolved HBV infection (defined as HBsAg-positive, or HBsAg-negative and anti-HBc-positive); (5) initiation of TACE/HAIC and TKIs within two weeks before or after the first dose of ICI; (6) receipt of at least one cycle of TACE/HAIC combined with at least one dose of a TKI and an ICI preoperatively; (7) concurrent receipt of prophylactic anti-HBV therapy during anti-tumor treatment; (8) Child-Pugh class A or B liver function; and (9) an Eastern Cooperative Oncology Group (ECOG) performance status score of 0–2.The exclusion criteria included: (1) presence of any other primary malignancy or extrahepatic metastases; (2) any prior anti-HCC treatment; (3) co-infection with other hepatotropic viruses or human immunodeficiency virus (HIV); (4) survival time of less than 3 months; (5) lack of HBV serological markers, HBV DNA monitoring, or imaging data during treatment; (6) history of organ or allogeneic bone marrow transplantation; (7) pregnancy or lactation; and (8) severe heart failure, uncontrolled diabetes, active infection, or other severe comorbidities. This study was approved by the Ethics Committee of Guangxi Medical University Cancer Hospital. The requirement for informed consent was waived due to the retrospective nature of the study. A total of 91 patients were ultimately included in the final analysis.

### 2.2 Conversion therapy

The conversion therapy regimen was tailored for each patient by a multidisciplinary team (MDT) based on their tumor status and liver function. The regimen consisted of transarterial interventional therapy (including TACE and HAIC) combined with a tyrosine kinase inhibitor (TKI) and a programmed cell death protein 1 (PD-1) inhibitor. Vascular interventional procedures (TACE and HAIC) were performed by interventional radiologists at our institution. Treatment with TKIs and PD-1 inhibitors was initiated within one week following the TACE or HAIC procedure, contingent upon the patient’s liver function recovery. The TKIs used in this study, consistent with the first-line treatment recommendations for advanced HCC in Chinese guidelines, included lenvatinib (8 mg daily for body weight <60 kg or 12 mg daily for body weight ≥60 kg), donafenib (0.2 g twice daily), sorafenib (400 mg twice daily), and apatinib (250 mg once daily). Bevacizumab was administered at 15 mg/kg every three weeks. The PD-1 inhibitors used were camrelizumab (200 mg intravenously IV every 2 weeks), tislelizumab (200 mg IV every 3 weeks), and sintilimab (200 mg IV every 3 weeks). The choice of specific agents was determined by the attending physician’s clinical judgment, the patient’s economic status, and personal preference. The dosage and frequency of all TKIs and PD-1 inhibitors were administered according to their respective package inserts.

### 2.3 Antiviral therapy

All patients were routinely screened for HBsAg, anti-HBs, HBeAg, anti-HBe, anti-HBc, and serum HBV DNA levels upon their initial admission. HBV DNA was quantified using a real-time quantitative polymerase chain reaction (qPCR) assay with a lower limit of detection of 20 IU/ml. Antiviral therapy was immediately initiated for patients with HBsAg-positive status or those who were HBsAg-negative but had detectable HBV DNA. The antiviral agents included entecavir (ETV, 0.5 mg/day), tenofovir disoproxil fumarate (TDF, 300 mg/day), and tenofovir alafenamide (TAF, 25 mg/day). Patients were allowed to make an informed choice regarding the specific drug based on their socioeconomic status and personal preference. For patients already receiving antiviral treatment prior to admission, their existing regimen was continued. Lifelong antiviral therapy was recommended for all patients with HBV-related HCC. To monitor for HBVr and ensure medication adherence, HBV DNA levels were measured every 6 weeks during conversion therapy, and medication intake was documented. Antiviral therapy was continued throughout the perioperative period, with HBV DNA levels checked on postoperative day 7. For patients who developed HBVr during treatment, their antiviral regimen was switched, although drug resistance testing was not performed.

### 2.4 Postoperative management and follow-up

Following surgery, patients were followed up every 2–3 months for the first two years and every 6 months thereafter. Monitoring included serum tumor markers (e.g., alpha-fetoprotein [AFP], protein induced by vitamin K absence-II [PIVKA-II]), HBV DNA, HBV serological markers, abdominal ultrasound, and contrast-enhanced computed tomography (CT) or magnetic resonance imaging (MRI).

### 2.5 Clinical and laboratory variables

Patient demographic characteristics and treatment histories were extracted from the electronic medical record system. Data on complete blood counts, blood biochemistry, AFP, HBV DNA, HBV serological markers, imaging studies, and tumor pathology were collected before and during anti-tumor treatment.

### 2.6 Outcome assessments

The primary endpoint was the incidence of HBVr, defined according to the Asian-Pacific Association for the Study of the Liver (APASL) clinical practice guidelines as one of the following: for patients with chronic HBV infection (HBsAg-positive), either (1) a ≥2 log_10_ IU/mL increase in HBV DNA level from baseline, or (2) an HBV DNA level >100 IU/mL in patients with previously undetectable baseline HBV DNA; for patients with resolved HBV infection (HBsAg-negative and anti-HBc-positive), either (1) HBsAg seroreversion (a change from HBsAg-negative to HBsAg-positive), or (2) a change from undetectable to detectable HBV DNA (Lau et al., 2021). Meeting any of these criteria signified an HBVr event. Secondary outcomes were overall survival (OS), progression-free survival (PFS), and loss to follow-up. OS was defined as the time from the initiation of the first treatment to cancer-related death or the last follow-up. PFS was defined as the time from surgical resection to disease progression, death from any cause, or the last follow-up. Tumor response was evaluated using the Response Evaluation Criteria in Solid Tumors (RECIST, version 1.1) and the HCC-specific modified RECIST (mRECIST) (Eisenhauer et al., 2009; Llovet and Lencioni, 2020). Tumor response was independently assessed by two radiologists who were blinded to the patients’ HBVr status.

### 2.7 Statistical analysis

Continuous variables were presented as mean ± standard deviation (SD) for normally distributed data and as median with interquartile range (IQR) for non-normally distributed data. Differences between groups were compared using the Student’s t-test or the Mann-Whitney U test, as appropriate. Categorical variables were described as numbers (n) and percentages (%) and were compared using the Chi-square (χ²) test or Fisher’s exact test. Univariate and multivariate logistic regression analyses were performed to identify risk factors for HBVr. Survival curves for PFS and OS were generated using the Kaplan-Meier method and compared with the log-rank test. The proportional hazards assumption for the Cox model was verified using the Schoenfeld residuals test. To identify independent prognostic factors for PFS and OS, univariate and multivariate analyses were conducted using the Cox proportional hazards model. A two-sided P-value of less than 0.05 was considered statistically significant. All statistical analyses were performed using SPSS software (version 25.0), and figures were generated with R software (version 4.4.2).

## 3 Results

### 3.1 Patient characteristics

**Figure 1:**
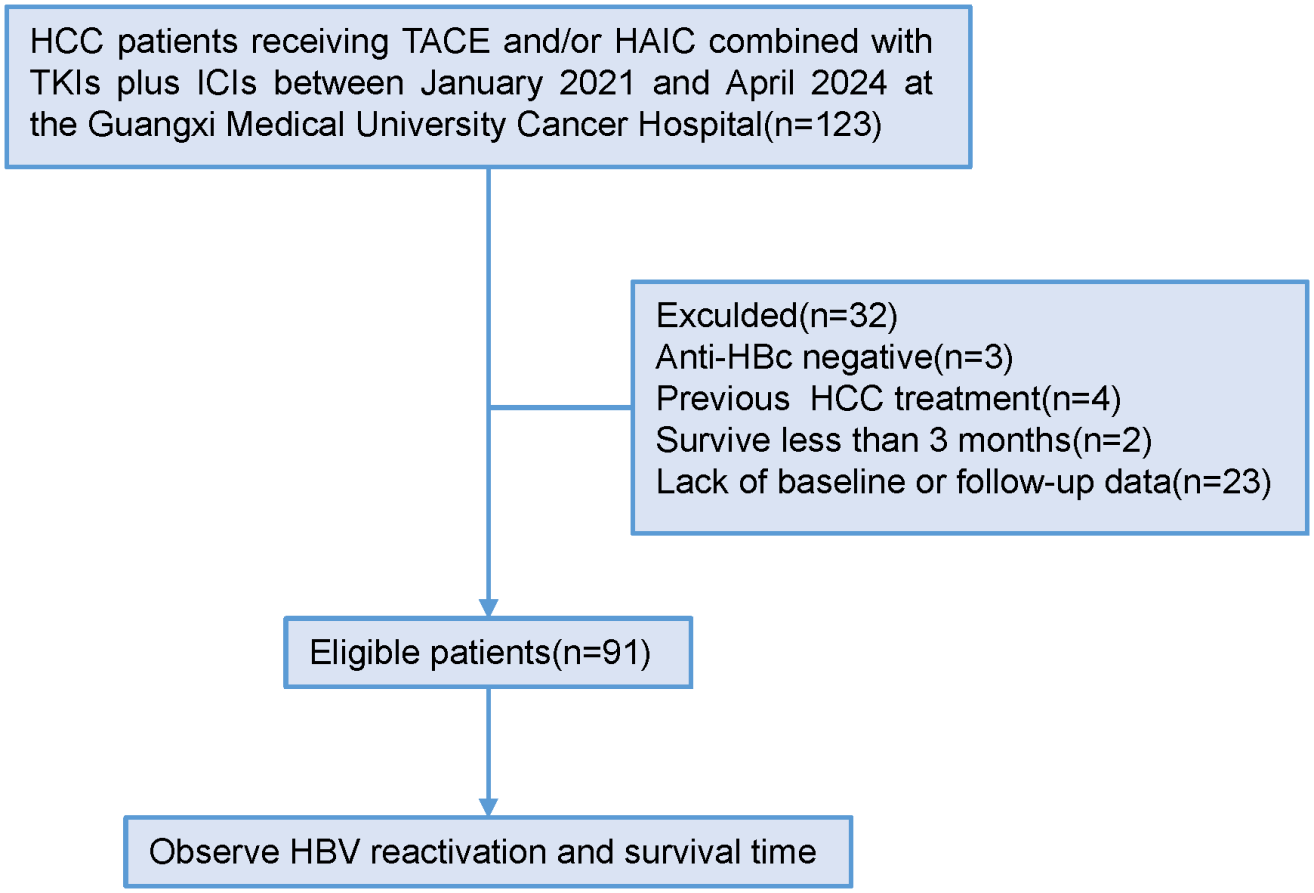
Patient enrollment and study flow. (Anti-HBc, antibody to hepatitis B core antigen; HAIC, hepatic artery infusion chemotherapy; HBV, hepatitis B virus; HCC, hepatocellular carcinoma; ICIs, immune checkpoint inhibitors; TACE, transarterial chemoembolization; TKIs, tyrosine kinase inhibitors).

From January 2021 to April 2024, a total of 123 patients with HCC who underwent tumor resection following conversion therapy with TACE and/or HAIC plus TKIs and ICIs were initially screened. Of these, 32 patients were excluded: 3 were anti-HBc negative, and 29 had missing baseline or follow-up data. Ultimately, 91 patients were eligible and included in the final analysis (**Figure 1**). The detailed baseline characteristics of the enrolled patients are summarized in **Table 1**. The ICIs administered included sintilimab, camrelizumab, or tislelizumab. The TKIs included sorafenib, lenvatinib, bevacizumab, donafenib, or apatinib. The patient age ranged from 27 to 72 years (median, 47 years), with a predominance of male patients (n=81, 89.0%). At baseline, 90 patients (98.9%) were HBsAg-positive, while one patient had occult HBV infection (HBsAg-negative, anti-HBc-positive, and HBV DNA-positive). A detectable baseline HBV DNA level (median, 221 IU/mL; range, 20–1,270,000 IU/mL) was present in 84 patients (92.3%), and 11 of these patients had a serum HBV DNA level >2000 IU/mL. All patients received antiviral therapy during conversion treatment, with agents including entecavir, tenofovir disoproxil fumarate, or tenofovir alafenamide. The cohort comprised 84 patients (92.3%) with Child-Pugh class A liver function and 7 (7.7%) with class B. According to the BCLC staging system, 24 patients (26.4%) were stage A, 27 (29.7%) were stage B, and 40 (44.0%) were stage C. Postoperative pathology revealed that 48 patients had a solitary tumor, and 43 had multiple tumors. The mean tumor size was 10.07 ± 4.18 cm. Portal vein tumor thrombus (PVTT) was present in 28 patients (30.8%), and microvascular invasion (MVI) was observed in 31 patients (34.1%). A pathological complete response (pCR) was achieved in 23 patients (25.3%).

**Table 1:** Baseline characteristics of patients with hepatocellular carcinoma.

Characteristics	Total (n=91)	HBV reactivation (n=17)	Non-reactivation (n=74)	P value
Age, years	47.00 (42.00,58.00)	45.00 (43.00,50.50)	48.00 (41.75,59.00)	0.280
Sex				0.778
Male	81 (89.0%)	15 (88.2%)	66 (89.2%)	
Female	10 (11.0%)	3 (11,8%)	8 (10.8%)	
Antiviral prophylaxis type				0.752
Entecavir	81 (89.0%)	16 (94.1%)	65 (87.8%)	
Tenofovir	10 (11.0%)	1 (5.9%)	9 (12.2%)	
HBsAg				0.340
Seropositive	89 (97.8%)	16 (94.1%)	73 (98.6%)	
Seronegative	2 (2.2%)	1 (5.9%)	1 (1.4%)	
HBeAg				0.690
Seropositive	36 (39.6%)	6 (35.3%)	30 (40.5%)	
Seronegative	55 (60.4%)	11 (64.7%)	44 (59.5%)	
ECOG PS				0.934
0	42 (46.2%)	8 (47.1%)	34 (45.9%)	
1-2	49 (53.8)	9 (52.9%)	40 (54.1%)	
Child Pugh grade				0.229
A	84 (92.3%)	14 (82.4%)	70 (94.6%)	
B	7 (7.7%)	3 (17.6%)	4 (5.4%)	
BCLC				0.478
A	24 (26.4%)	5 (29.4%)	19 (25.7%)	
B	27 (29.7%)	3 (17.6%)	24 (32.4%)	
C	40 (44.0%)	9 (52.9%)	31 (41.9%)	
HBV DNA, IU/ml				
Undetectable	7 (7.7%)	3 (17.6%)	4 (5.4%)	0.229
Detectable	84 (92.3)	14 (82.4%)	70 (94.6%)	
>2000	11 (12.1%)	3 (17.6%)	8 (10.8%)	0.713
≤2000	80 (87.9%)	14 (82.4%)	66 (89.2%)	
Median baseline HBV DNA (range), IU/mL	221.0 (0-3110000)	455.0 (0-3110000)	180.5 (0-30300)	
ALT, U/L				0.067
>40	46 (50.5%)	12 (70.6%)	34 (45.9%)	
≤ 40	45 (49.5%)	5 (29.4%)	40 (54.1%)	
TBil, mmol/L	16.61 (10.30,20.30)	17.28 (11.30,23.45)	16.46 (10.30,19.88)	0.521
ALB, g/L	38.05 (± 4.36)	37.44 (± 4.33)	38.19 (± 4.39)	0.524
ALBI grade				0.582
I	32 (35.2%)	5 (29.4%)	27 (36.5%)	
II	59 (64.8%)	12 (70.6%)	47 (63.5%)	
AFP, ng/mL				0.675
AFP≥1000	44 (48.4%)	9 (52.9%)	35 (47.3%)	
AFP<1000	47 (51.6%)	8 (47.1%)	39 (52.7%)	
WBC, ×109/L	6.45 (5.01,7.50)	6.95 (5.33,7.93)	6.19 (4.97,7.36)	0.261
Hemoglobin, g/L	136.03 (± 21.19)	127.94 (± 24.43)	137.89 (± 20.10)	0.081
Platelet, ×109/L	214 (170,278)	201 (153,293)	216 (170,271)	0.867
Cirrhosis				0.641
Yes	74 (81.3%)	15 (88.2%)	59 (79.7%)	
No	17 (18.7%)	2 (11.8%)	15 (20.3%)	
Tumor diameter (cm)	10.07 (± 4.18)	10.94 (± 5.41)	9.88 (± 3.87)	0.349
≥10cm	43 (47.3%)	8 (47.1%)	35 (47.3%)	0.986
<10cm	48 (52.7%)	9 (52.9%)	39 (52.7%)	
Tumor number				0.578
Single	48 (52.7%)	10 (58.8%)	38 (51.4%)	
Multiple	43 (47.3%)	7 (41.2%)	36 (48.6%)	
PVTT				0.893
Yes	28 (30.8%)	5 (29.4%)	23 (31.1%)	
No	63 (69.2%)	12 (70.6%)	51 (68.9%)	
MVI				0.560
Yes	18	2 (11.8%)	16 (21.6%)	
No	73	15 (88.2%)	58 (78.4%)	
Vascular invasion				0.493
Yes	31 (34.1%)	7 (41.2%)	24 (32.4%)	
No	60 (65.9%)	10 (58.8%)	50 (67.6%)	
pCR				0.083
Yes	23 (25.3%)	1 (5.9%)	22 (29.7%)	
No	68 (74.7%)	16 (94.1%)	52 (70.3%)	
Types of TKIs				0.342
Lenvatinib	77 (84.6%)	16 (94.1%)	61 (82.4%)	
Donafenib	8 (8.8%)	1 (5.9%)	7 (9.5%)	
Apatinib	3 (3.3%)	0 (0%)	3 (4.1%)	
Sorafanib	1 (1.1%)	0 (0%)	1 (1.4%)	
Bevacizumab	1 (1.1%)	0 (0%)	1 (1.4%)	
Donafenib+ Lenvatinib	1 (1.1%)	0 (0%)	1 (1.4%)	
Types of ICIs				0.743
Camrelizumab	51 (56.0%)	10 (58.8%)	41 (55.4%)	
Tislelizumab	32 (35.2%)	6 (35.3%)	26 (35.1%)	
Sintilimab	4 (4.4%)	0 (0%)	4 (5.4%)	
Tislelizumab+ Sintilimab	2 (2.2%)	1 (5.9%)	1 (1.4%)	
Tislelizumab+ Camrelizumab	1 (1.1%)	0 (0%)	1 (1.4%)	
Sintilimab+ Camrelizumab	1 (1.1%)	0 (0%)	1 (1.4%)	
Types of interventional therapy				0.938
HAIC	19 (20.9%)	4 (23.5%)	15 (20.3%)	
TACE	65 (71.4%)	11 (64.7%)	54 (73.0%)	
HAIC+TACE	7 (7.7%)	2 (11.8%)	5 (6.8%)	

HBV, hepatitis B virus; HBsAg, hepatitis B surface antigen; HBeAg, hepatitis B e antigen; AFP, alpha-fetoprotein; ALT, alanine aminotransferase; AST, aspartate aminotransferase; TBil, total bilirubin; WBC, white blood cell; BCLC, Barcelona Clinic Liver Cancer; ECOG PS, Eastern Cooperative Oncology Group performance status; ALBI grade, Albumin-Bilirubin grade; MVI, microvascular invasion; PVTT, portal vein tumor thrombosis; DNA, deoxyribonucleic acid; HAIC, hepatic arterial infusion chemotherapy; PD-1 inhibitors, programmed death receptor-1 inhibitors; TACE, transarterial chemoembolization; TKIs, tyrosine kinase inhibitors; pCR, pathological complete response.

### 3.2 HBV reactivation

**Figure 2:**
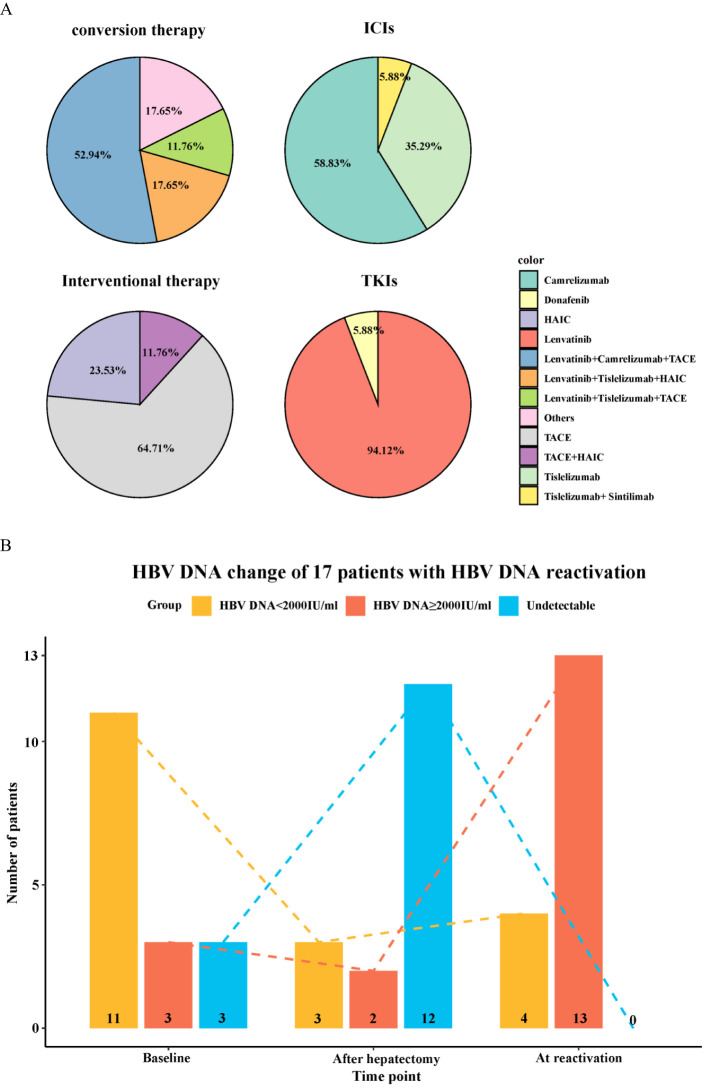
Characteristics of patients with hepatitis B virus reactivation. **(A)** Baseline demographics and clinical characteristics of the 17 patients who experienced HBVr. **(B)** Changes in HBV DNA levels over time in the 17 patients with HBVr.

Among the 91 enrolled patients, HBVr occurred in a total of 17 patients (18.7%), with a median time to reactivation of 3 months (range, 1–10 months). Additionally, 19 patients experienced a certain degree of increase in viral load that did not meet the criteria for reactivation. Detailed characteristics of the 17 patients with HBVr are presented in **Table 2** and **Figure 2**. Of these 17 patients, 15 were male. Three patients had undetectable HBV DNA at baseline. Among those with detectable baseline DNA, 12 achieved virological suppression during preoperative antiviral therapy. One patient was HBsAg-negative at baseline. At the onset of HBVr, the median HBV DNA level was 495 IU/mL (range, 109–6,710,000 IU/mL). All 17 patients with reactivation had received antiviral therapy since their initial diagnosis of hepatitis B, with 16 of them taking entecavir. The incidence of HBVr was 16.7% (14/84) in patients with detectable baseline HBV DNA and 42.9% (3/7) in those with undetectable baseline HBV DNA.

**Table 2:** Characteristics of patients with hepatitis B virus reactivation.

Patient characteristics					Baseline			At reactivation			
NO	Age/Sex	Types of ICIs	Types of TKIs	Types of interventional therapy	HBsAg	HBV DNA IU/ml	Antiviral treatment	Intervals (months)	HBsAg	HBV DNA IU/ml	Antiviral treatment
1	39/M	Camrelizumab	Lenvatinib	TACE	+	1610	Entecavir	4	+	386	Entecavir
2	46/M	Tislelizumab	Lenvatinib	TACE	+	Undetectable	Entecavir	2	+	180	Entecavir
3	44/M	Tislelizumab	Lenvatinib	HAIC+TACE	+	389	Entecavir	3	+	150	Entecavir
4	47/M	Tislelizumab	Donafenib	HAIC	+	833	Tenofovir	3	+	145	Tenofovir
5	58/M	Camrelizumab	Lenvatinib	HAIC+TACE	+	3670	Entecavir	2	+	362	Entecavir
6	45/M	Tislelizumab	Lenvatinib	HAIC	+	566	Entecavir	8	+	109	Entecavir
7	60/M	Camrelizumab	Lenvatinib	TACE	+	Undetectable	Entecavir	14	+	6710000	Entecavir
8	44/F	Camrelizumab	Lenvatinib	TACE	+	137	Entecavir	10	+	6790	Entecavir
9	42/M	Camrelizumab	Lenvatinib	TACE	–	455	Entecavir	4	+	706	Entecavir
10	45/M	Camrelizumab	Lenvatinib	TACE	+	857	Entecavir	1	+	1500	Entecavir
11	35/M	Camrelizumab	Lenvatinib	TACE	+	47	Entecavir	2	+	526	Entecavir
12	61/M	Camrelizumab	Lenvatinib	TACE	+	Undetectable	Entecavir	2	+	135	Entecavir
13	34/M	Camrelizumab	Lenvatinib	TACE	+	722	Entecavir	2	+	151	Entecavir
14	50/M	Camrelizumab	Lenvatinib	TACE	+	2300	Entecavir	3	+	11500	Entecavir
15	51/M	Tislelizumab	Lenvatinib	HAIC	+	109	Entecavir	4	+	495	Entecavir
16	44/F	Tislelizumab	Lenvatinib	TACE	+	442	Entecavir	5	+	79300	Entecavir
17	45/M	Tislelizumab+ Sintilimab	Lenvatinib	HAIC	+	3110000	Entecavir	4	+	737	Entecavir

DNA, deoxyribonucleic acid; F, female; HAIC, hepatic artery infusion chemotherapy; HBV, hepatitis B virus; HBsAg, hepatitis B surface antigen; M, male; TACE, transarterial chemoembolization.

### 3.3 Patterns of HBV reactivation

Among the 17 patients who experienced HBVr, a notable pattern was observed in 12 individuals who had initially achieved virological suppression (from detectable to undetectable) during preoperative antiviral therapy but subsequently showed detectable HBV DNA postoperatively. Furthermore, one HBsAg-negative patient experienced HBsAg seroreversion after surgery. All these patients were receiving ETV during the treatment period and remained HBsAg-positive post-reactivation, except for the single case of seroreversion.

### 3.4 Univariate and multivariable analyses for HBV reactivation

The results of the univariate and multivariable logistic regression analyses for HBVr are shown in **Table 3**. Both analyses consistently identified baseline HBV DNA ≥2000 IU/mL as the sole independent risk factor for HBVr (OR 3.939, 95% CI 1.169–13.272; P = 0.027).

**Table 3:** Univariate and multivariate logistic regression analysis of risk factors for hepatitis B virus reactivation.

	Univariate		Multivariate	
	OR(95%CI)	P value	OR(95%CI)	P value
Age (≥50 years)	0.490(0.157-1.531)	0.220		
Sex(female)	0.909(0.175-4.723)	0.910		
BCLC (C)	1.560(0.541-4.497)	0.410		
ECOG (≥1)	0.956(0.332-2.750)	0.934		
HBV DNA (detectable)	0.267(0.054-1.325)	0.106		
HBV DNA (≥2000 IU/ml)	3.939(1.169-13.272)	0.027	3.939(1.169-13.272)	0.027
Child Pugh score (B)	3.750(0.755-18.633)	0.106		
Tumor diameter (≥10cm)	0.990(0.345-2.848)	0.986		
Tumor number (multiple)	0.739(0.254-2.150)	0.579		
PVTT (yes)	0.924(0.291-2.928)	0.893		
MVI (yes)	0.483(0.100-2.337)	0.366		
AFP (≥400ng/ml)	0.905(0.315-2.606)	0.854		
AFP (≥200 ng/ml)	0.870(0.297-2.545)	0.799		
ALT (≥50IU/L)	1.552(0.525-4.589)	0.427		
Albumin (≥35 g/L)	0.771(0.239-2.487)	0.664		
TBil (≥17.1mmol/L)	0.971(0.333-2.832)	0.957		
WBC (≥11*109/L)	0.999(0-0)	1		
Liver cirrhosis (yes)	1.907(0.393-9.262)	0.423		
Antiviral prophylaxis type	2.215(0.261-18.776)	0.466		
Types of TKIs (Lenvatinib)	3.410(0.415-28.045)	0.254		
Types of ICIs (Camrelizumab)	1.150(0.395-3.349)	0.798		
Types of interventional therapy (multiple)	1.840(0.325-10.402)	0.490		

AFP, alpha-fetoprotein; ALBI grade, Albumin-Bilirubin grade; ALT, alanine aminotransferase; AST, aspartate aminotransferase; BCLC, Barcelona Clinic Liver Cancer; DNA, deoxyribonucleic acid; ECOG PS, Eastern Cooperative Oncology Group performance status; HBeAg, hepatitis B e antigen; HBsAg, hepatitis B surface antigen; HBV, hepatitis B virus; MVI, microvascular invasion; PD-1 inhibitors, programmed death receptor-1 inhibitors; pCR, pathological complete response; PVTT, portal vein tumor thrombosis; TBil, total bilirubin; TKIs, tyrosine kinase inhibitors; WBC, white blood cell.

### 3.5 Patient prognosis

**Figure 3:**
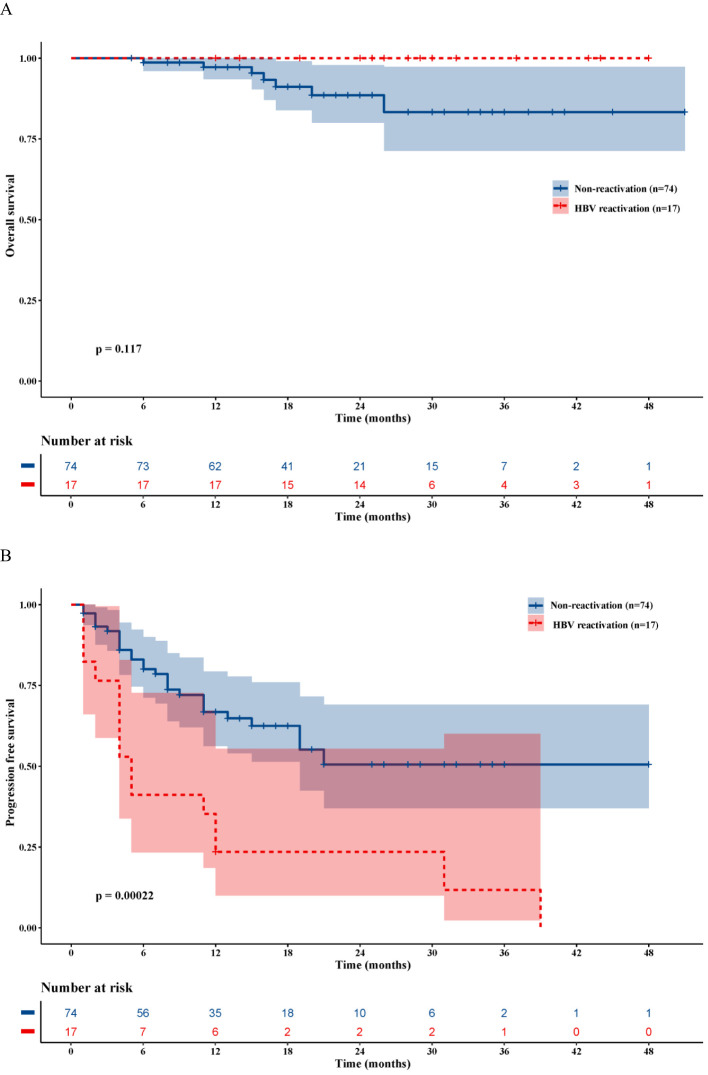
Kaplan-Meier survival analysis. **(A)** Overall survival curves for patients with and without HBVr. **(B)** Progression-free survival curves for patients with and without HBVr.

The median OS and PFS for the entire cohort of 91 patients were 47.0 months and 23.6 months, respectively (**Supplementary Figure S1**). The median follow-up time was 28.8 months in the HBVr group and 20.6 months in the non-reactivation group. No deaths were observed in the HBVr group during the follow-up period. The median OS was not reached in the HBVr group, compared to 45.6 months (95% CI 41.7–49.5) in the non-reactivation group (P = 0.117) (**Figure 3A**). However, the median PFS was significantly shorter in the HBVr group than in the non-reactivation group (12.1 months [95% CI 5.5–18.7] *vs*. 29.2 months [95% CI 23.6–34.7]; P < 0.001) (**Figure 3B**). These findings suggest that patients in the HBVr group had a higher risk of disease recurrence.

### 3.6 Univariate and multivariable analyses for PFS and OS

**Figure 4:**
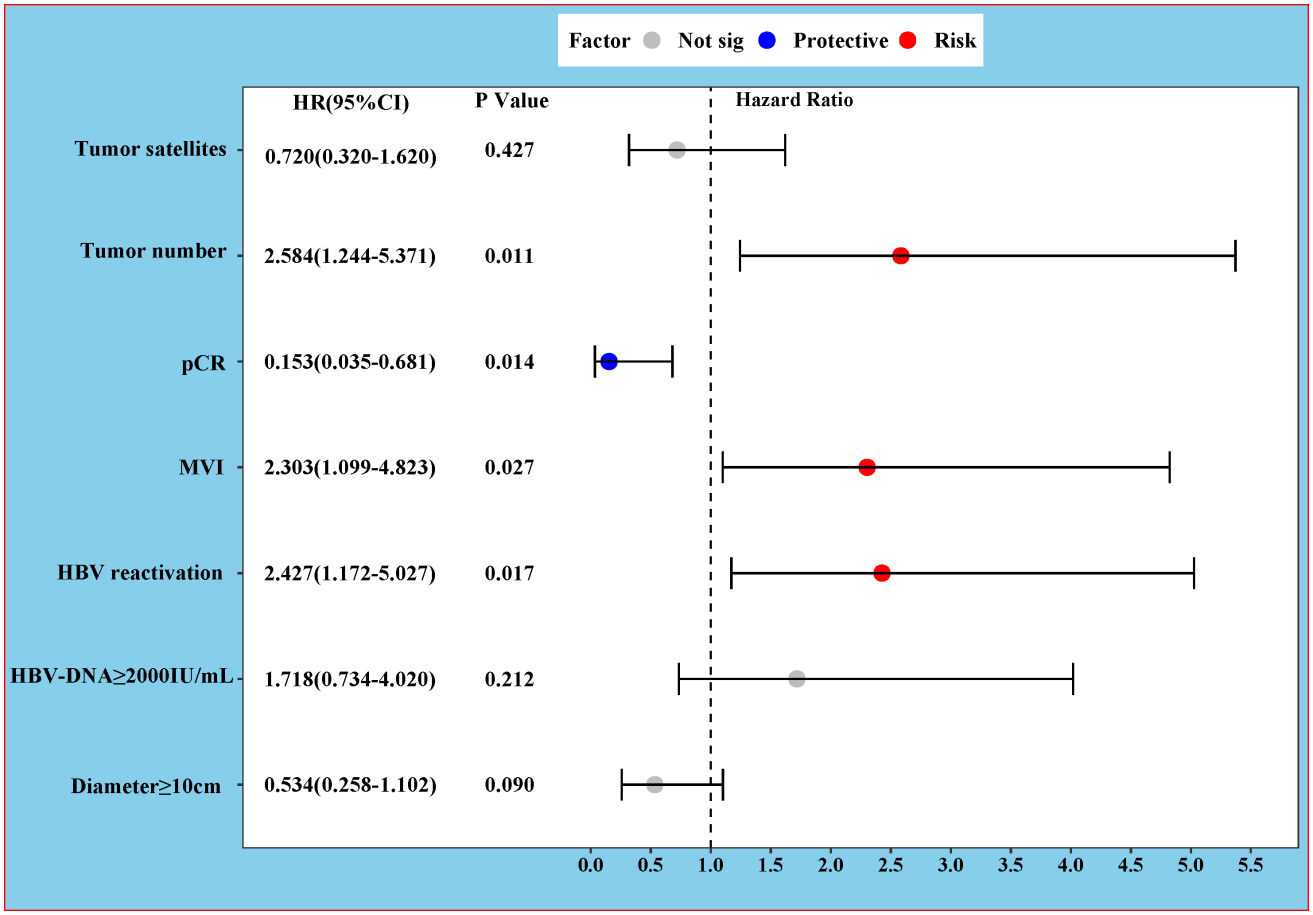
Forest Plot of Hazard Ratios for Progression-Free Survival.

The results of the univariate and multivariable Cox regression analyses for PFS and OS are presented in **Table 4**. For PFS, univariate analysis identified several significant risk factors: multiple tumors (HR 2.418, 95% CI 1.283–4.557; P = 0.006), tumor diameter ≥10 cm (HR 2.433, 95% CI 1.256–4.714; P = 0.009), baseline HBV DNA ≥2000 IU/mL (HR 2.385, 95% CI 1.227–4.636; P = 0.010), HBVr (HR 3.085, 95% CI 1.623–5.863; P = 0.001), presence of satellite nodules (HR 2.117, 95% CI 1.058–4.236; P = 0.034), and MVI (HR 4.804, 95% CI 2.506–9.210; P < 0.001). pCR was a significant protective factor (HR 0.103, 95% CI 0.025–0.428; P = 0.002). In the multivariable analysis for PFS, multiple tumors (HR 2.584, 95% CI 1.244–5.371; P = 0.011), HBVr (HR 2.427, 95% CI 1.172–5.027; P = 0.017), and MVI (HR 2.303, 95% CI 1.099–4.823; P = 0.027) remained independent risk factors. pCR remained an independent protective factor (HR 0.153, 95% CI 0.035–0.681; P = 0.014) (**Figure 4**). For OS, both univariate and multivariable analyses identified baseline HBV DNA ≥2000 IU/mL as the sole independent risk factor (HR 6.549, 95% CI 1.458–29.408; P = 0.014). The proportional hazards assumption was met for all Cox models, as verified by Schoenfeld residual tests (P > 0.05 for all variables).

**Table 4:** Univariate and multivariate Cox regression analysis of independent predictors for progression-free survival and overall survival.

Variables	Progression-free survival						Overall survival					
	Univariate			Multivariate			Univariate			Multivariate		
	HR	95% CI	P	HR	95% CI	P	HR	95% CI	P	HR	95% CI	P
Age, years≥50:<50	0.662	0.352-1.246	0.201				1.115	0.249-4.989	0.887			
Sex, male: female	1.927	0.585-6.347	0.281				23.528	0-1388629.090	0.573			
AFP, μg/L ≥ 400, yes: no	1.061	0.577-1.953	0.848				2.437	0.469-12.653	0.289			
Tumor number, multiple: single	2.418	1.283-4.557	0.006	2.584	1.244-5.371	0.011	2.822	0.546-14.587	0.216			
Liver cirrhosis, yes: no	1.069	0.492-2.323	0.867				0.476	0.092-2.470	0.377			
Diameter, cm, ≥10:< 10	0.411	0.212-0.797	0.009	0.534	0.258-1.102	0.090	0.703	0.157-3.151	0.646			
BCLC staging, C: AB	1.271	0.693-2.331	0.438				6.552	0.787-54.538	0.082			
ECOG, ≥1:0	0.758	0.284-2.022	0.580				0.699	0.258-1.889	0.480			
Total bilirubin, μmol/L	0.999	0.957-1.044	0.981				0.922	0.804-1.058	0.247			
Albumin, g/L	1.012	0.944-1.084	0.743				0.969	0.827-1.135	0.692			
Platelets, 10^9^/L	0.999	0.996-1.003	0.702				0.999	0.990-1.007	0.778			
Prothrombin time, s	1.120	0.885-1.417	0.347				0.916	0.550-1.525	0.736			
ALT, U/L,>40:≤40	1.224	0.664-2.258	0.517				0.149	0.018-1.243	0.079			
HBV-DNA, IU/mL, ≥ 2000: < 2000	2.385	1.227-4.636	0.010	1.718	0.734-4.020	0.212	6.549	1.458-29.408	0.014	9.825	2.114-45.667	0.004
HBV reactivation, yes: no	3.085	1.623-5.863	0.001	2.427	1.172-5.027	0.017	0.030	0-42.929	0.344			
History of alcoholism, yes: no	1.035	0.557-1.924	0.913				0.524	0.102-2.706	0.441			
Interventional therapy, Entecavir: Tenofovir	1.687	0.520-5.477	0.384				0.191	0.036-1.002	0.050			
PVTT, yes: no	1.081	0.573-2.042	0.810				2.486	0.555-11.144	0.234			
Large vascular invasion, yes: no	1.177	0.634-2.187	0.606				1.166	0.260-5.236	0.841			
Tumor satellites, yes: no	2.117	1.058-4.236	0.034	0.720	0.320-1.620	0.427	1.544	0.295-8.077	0.607			
pCR, yes: no	0.103	0.025-0.428	0.002	0.153	0.035-0.681	0.014	0.029	0-28.133	0.312			
MVI, yes: no	4.804	2.506-9.210	0.000	2.303	1.099-4.823	0.027	2.450	0.470-12.759	0.287			

AFP, alpha-fetoprotein; ALBI grade, Albumin-Bilirubin grade; ALT, alanine aminotransferase; AST, aspartate aminotransferase; BCLC, Barcelona Clinic Liver Cancer; DNA, deoxyribonucleic acid; ECOG PS, Eastern Cooperative Oncology Group performance status; HBeAg, hepatitis B e antigen; HBsAg, hepatitis B surface antigen; HBV, hepatitis B virus; MVI, microvascular invasion; pCR, pathological complete response; PD-1 inhibitors, programmed death receptor-1 inhibitors; PVTT, portal vein tumor thrombosis; TBil, total bilirubin; TKIs, tyrosine kinase inhibitors; WBC, white blood cell.

## 4 Discussion

This retrospective study is the first to elucidate the incidence of HBVr and evaluate its prognostic impact in patients with HBV-related HCC who underwent surgical resection following conversion therapy with interventional treatment, TKIs, and ICIs. We found that 17 (18.7%) patients experienced HBVr. Compared to the non-reactivation group, the HBVr group had a significantly shorter PFS, although no significant difference in OS was observed. The lack of a statistically significant OS difference may be attributable to the relatively small sample size, rendering the analysis underpowered. Furthermore, we identified a baseline HBV DNA level ≥2000 IU/mL as an independent risk factor for HBVr. For prognosis, multiple tumors, MVI, and HBVr were independent risk factors for tumor recurrence, whereas pCR was an independent protective factor. A baseline HBV DNA level ≥2000 IU/mL was the sole independent predictor of mortality.

Anti-tumor therapies, including surgery, TACE, HAIC, TKIs, and ICIs, have all been associated with HBVr. In our cohort, HBVr was observed in 18.7% of patients. This incidence is notably higher than that reported in studies of patients receiving combination therapies without subsequent surgery. For instance, the reported HBVr rate in HCC patients undergoing surgical resection with prophylactic antiviral therapy is typically between 1% and 5% (Papatheodoridi et al., 2022). In patients treated with TACE plus targeted and immune therapies, the HBVr rate was 10.1% (Shen et al., 2023), while for those on HAIC plus targeted and immune therapies, it was 7.5% (Yang et al., 2024). The primary cause of HBVr is an imbalance between the host’s immune response and viral replication. Surgical resection itself is a known risk factor for HBVr in HBsAg-positive patients, largely due to the surgical stress response, which can impair the host’s immune status, particularly in cases of concurrent infection or decompensated liver function (Papatheodoridi et al., 2022). The metabolic and immunological stress induced by hepatectomy, along with the acute release of stress hormones and cytokines, creates a transient window of immunosuppression, rendering patients susceptible to HBVr Burpee (Burpee et al., 2002). Moreover, partial hepatectomy can enhance viral replication due to immunosuppression from blood transfusions and ischemia-reperfusion injury (Huang et al., 2012). It is plausible that the combination of immunosuppression from conversion therapy and the subsequent surgical stress synergistically exacerbates immune dysfunction, leading to a higher HBVr rate than either treatment modality alone (Liu et al., 2021). Combination therapy is associated with an increased risk of HBVr. Indeed, several recent studies have identified combination therapy as an independent risk factor for this event (Lei et al., 2023; Wang et al., 2024). However, the underlying mechanisms for the elevated risk of HBV reactivation in patients undergoing surgical resection after conversion therapy remain to be fully elucidated. We speculate that this may be attributed to the incomplete recovery of host immune function following conversion therapy. This pre-existing immune compromise, when compounded by surgical stress, could lead to further immunosuppression, thereby resulting in a higher incidence of HBV reactivation.

In our study, HBVr occurred in 27.3% (3/11) of patients with baseline HBV DNA ≥2000 IU/mL and 17.5% (14/80) of those with levels <2000 IU/mL. Furthermore, multivariate analysis identified a baseline HBV DNA level of ≥2000 IU/mL as an independent risk factor for HBV reactivation. These findings are consistent with those of several previous reports. For instance, a study on HBV reactivation after radiofrequency ablation in patients with HCC reported that an HBV DNA level ≥2000 IU/mL was a significant risk factor (Liu et al., 2023). The observation that patients with higher HBV DNA levels are more prone to reactivation than those with lower levels has been well-documented in multiple studies (Cholongitas et al., 2018; Wang et al., 2021; Shen et al., 2023). However, some studies have reported no significant association between baseline HBV DNA levels and HBV reactivation in the context of combination therapy (He et al., 2021; Yang et al., 2024). This discrepancy may be attributable to the subsequent surgical intervention following conversion therapy, which could further alter both local and systemic immune statuses. This suggests that different treatment modalities may confer varying risks of HBV reactivation. Despite all patients receiving antiviral prophylaxis, HBVr still occurred. One possible explanation is the development of antiviral resistance resulting from prior treatments (Tenney et al., 2009; Guo et al., 2018). Another potential reason could be the disruption of antiviral therapy due to poor patient adherence, where patients fail to take their medication regularly. This phenomenon is not uncommon and has been documented in numerous studies (Jang, 2014; Shen et al., 2023; Yang et al., 2024). Our results showed no definitive link between the choice of specific interventional, targeted, or immune agents and HBVr risk. This suggests that the profound immunological insult from surgery may overshadow the differential effects of various conversion regimens. Therefore, for patients with high baseline HBV DNA levels, adopting a more potent antiviral strategy perioperatively may be warranted.

Histopathological features of the tumor were strongly associated with PFS. Our analysis confirmed that multiple tumors and MVI are independent risk factors for postoperative recurrence, while pCR is a strong protective factor. These findings align with established literature, where tumor size, multifocality, satellite nodules, and MVI have been consistently identified as predictors of a higher recurrence risk (Imamura et al., 2003; Sala et al., 2004; Ishizawa et al., 2008; Schiffman et al., 2010; Fuks et al., 2012; Li et al., 2013). Interestingly, we did not find a significant association between tumor size or satellite nodules and recurrence, which might be due to the larger tumor burden in our cohort compared to previous studies, or perhaps the preoperative conversion therapy altered the biological characteristics of the tumors.

Crucially, our study identified HBVr as an independent risk factor for postoperative tumor recurrence, corroborating findings from other recent studies (Lei et al., 2023; Yang et al., 2024). This association likely reflects a vicious cycle between the virus and the tumor. On one hand, HBVr involves a surge in viral replication and antigen release, triggering a robust inflammatory response. This chronic inflammation, often involving the activation of NF-κB and MAPK signaling pathways, creates a microenvironment conducive to hepatocellular mutagenesis and epigenetic alterations, thereby promoting HCC progression (Feitelson et al., 2022; Sivasudhan et al., 2022). This inflammatory state can also foster an immunosuppressive milieu by recruiting regulatory T cells (Tregs) and promoting anti-inflammatory cytokines, which impair immune surveillance (Chekol Abebe et al., 2021). On the other hand, the immunosuppressive environment created by tumor progression can facilitate HBVr. Tumors can upregulate immunosuppressive molecules like TGF-β and PD-L1 and pro-angiogenic factors like VEGF, which collectively inhibit T cell and NK cell function and promote the accumulation of Tregs and myeloid-derived suppressor cells (MDSCs) (Wang et al., 2011; Kalluri, 2016; Yang et al., 2018). Given this interplay, we propose that a more aggressive antiviral strategy should be considered during the perioperative period to minimize the risk of HBVr and, consequently, reduce the likelihood of tumor recurrence. However, the optimal timing to de-escalate back to a standard antiviral regimen postoperatively requires further investigation.

A high HBV DNA load is known to correlate with poor prognosis in HCC patients. In our study, a baseline HBV DNA level ≥2000 IU/mL was the sole independent risk factor for OS, although the wide confidence interval (HR 6.549, 95% CI 1.458–29.408) suggests that this finding may be limited by the sample size. This association has been repeatedly documented in the literature (Yu and Kim, 2014; Sun et al., 2021; Yang et al., 2024). We also observed a high HBVr rate (42.9%) among patients with undetectable baseline HBV DNA. This underscores the persistence of cccDNA in hepatocytes, which serves as a template for reactivation even when serum DNA is suppressed by nucleos(t)ide analogues (NAs) (Xia and Guo, 2020). Theoretically, even a single copy of cccDNA can lead to viral rebound and trigger chronic inflammation, perpetuating the malignant cycle of HBV and HCC (Shi and Zheng, 2020). Contrary to some previous reports, we did not find an association between tumor pathology or HBVr and OS. This could be due to the heterogeneity of post-recurrence treatments received by patients in our cohort, which would significantly influence survival outcomes. The impact of post-recurrence therapies in this specific patient population warrants further investigation.

This study has several limitations. First, as a single-center retrospective study with a relatively small sample size, selection bias cannot be ruled out. Second, we did not perform mechanistic studies to elucidate the biological links between HBVr and the combined treatment modality. Basic research is needed to explore these mechanisms. Finally, the screening intervals for HBV DNA and serological markers were not standardized, which may have led to delays in detecting some endpoint events. Therefore, large-scale, prospective, multicenter, randomized controlled trials are warranted to validate our conclusions.

## 5 Conclusion

This study indicates that in patients with HBV-related HCC undergoing surgery after conversion therapy, a high baseline HBV DNA level may lead to HBV reactivation and adversely affect long-term survival. Patients who experience HBV reactivation have a higher risk of recurrence than those who do not. Therefore, antiviral therapy and HBV DNA monitoring should be administered to patients with HBV-related HCC during conversion therapy and throughout the perioperative period.

## Data Availability

The datasets presented in this study can be found in online repositories. The names of the repository/repositories and accession number(s) can be found in the article/**Supplementary Material**.
