# Supplementary figures and images for: Postoperative hepatitis B virus reactivation and its impact on survival in HBV-related hepatocellular carcinoma patients undergoing conversion therapy with interventional therapy combined with tyrosine kinase inhibitors and immune checkpoint inhibitors
Source: Front Cell Infect Microbiol. 2025 Jul 17;15:1598193. doi: 10.3389/fcimb.2025.1598193 (PMC12310596; doi:10.3389/fcimb.2025.1598193)

Supplementary Material

**Supplementary Figure 1.**

**
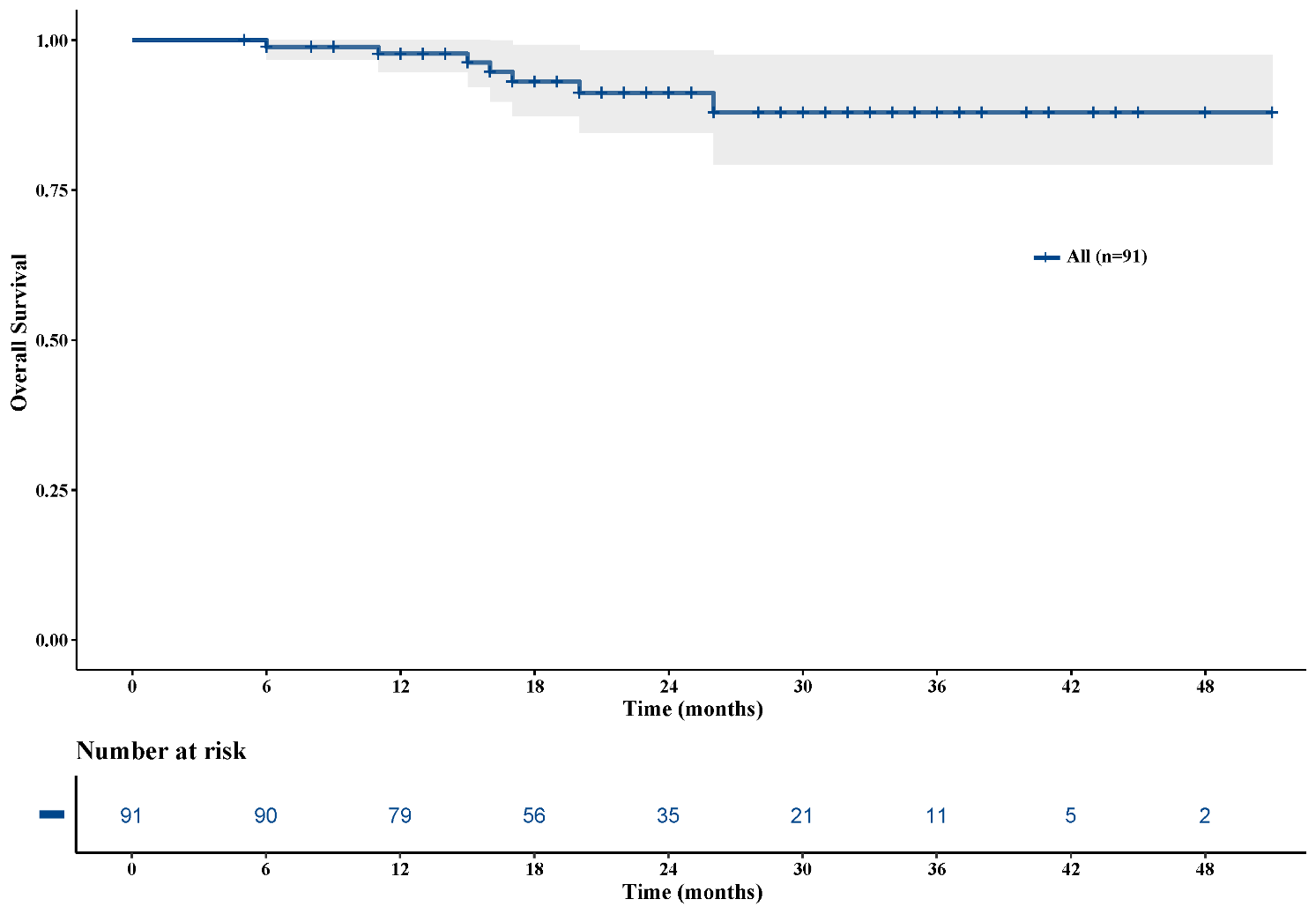
**

**Supplementary Figure 2.**


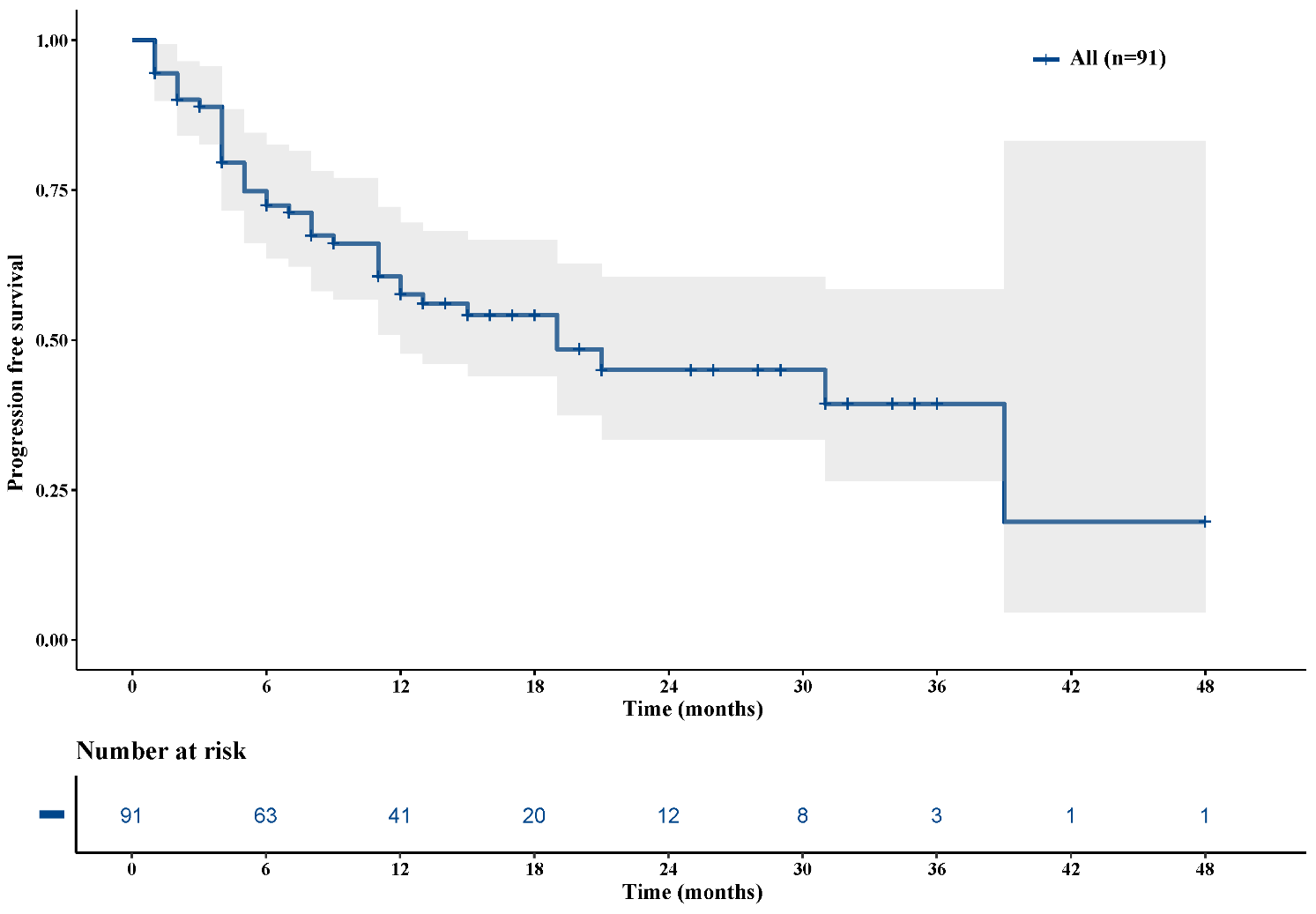

Supplement: Supplementary file 1 [file Table1.docx]
